# EMPOWERING AND ADDRESSING AGE-RELATED CONCERNS AMONG OLDER WOMEN LIVING WITH HIV

**DOI:** 10.1093/geroni/igad104.0446

**Published:** 2023-12-21

**Authors:** Thi Vu, Marielle Quinn, Sarah Valeika, Joan Monin

**Affiliations:** Yale University School of Public Health, New Haven, Connecticut, United States; Yale University School of Public Health, New Haven, Connecticut, United States; Yale University, New Haven, Connecticut, United States; Yale University School of Public Health, New Haven, Connecticut, United States

## Abstract

There are currently over 100,000 older (ages 50+ years) women living with HIV/AIDs in the United States. Older women living with HIV (OWLH) face unique psychosocial challenges with HIV prevention and care, including intimate partner violence, gender discrimination, ageism, and caregiving responsibilities. These factors can impede self-care and contribute to increased mental, emotional, and physical burdens. Little is known about the resources OWLH draw upon to help manage psychosocial stressors and their perspectives of aging with HIV. This qualitative study identified sources of strength and self-care among OWLH, as well as their concerns about aging with HIV. We recruited participants through HIV clinics and community support groups. We conducted one-on-one, semi-structured in-depth phone interviews with OWLH. Interviews were recorded and transcribed verbatim. Following a Grounded Theory approach, two coders performed open coding then thematic coding. Participants (N = 23) were on average 60 years old (range 51 – 68) and had been living with HIV for an average of 23.7 years. We found that a desire to self-educate, community activism, and engagement with other OWLH reduced participants’ internalized stigma and were sources of empowerment. However, concerns about aging with HIV persists, including pervasive HIV stigma, comorbidities, shrinking caregiver networks, long-term effects of treatment, and lack of HIV research and programs focusing on older women. These findings suggest that while OWLH are empowered agents in their own HIV care journey, structural interventions (e.g., inclusion of older women in HIV trials) are still needed to address the unique concerns of this population.

